# Group I Paks as therapeutic targets in *NF2*-deficient meningioma

**DOI:** 10.18632/oncotarget.2810

**Published:** 2015-01-24

**Authors:** Hoi-Yee Chow, Biao Dong, Sergio G. Duron, David A. Campbell, Christy C. Ong, Klaus P. Hoeflich, Long-Sheng Chang, D. Bradley Welling, Zeng-jie Yang, Jonathan Chernoff

**Affiliations:** ^1^ Cancer Biology Program, Fox Chase Cancer Center, Philadelphia, Pennsylvania 19111, USA; ^2^ Department of Microbiology and Immunology, Sol Sherry Thrombosis Research Center, Temple University, Philadelphia, Pennsylvania 19104, USA; ^3^ Afraxis Inc., La Jolla, California 92037, USA; ^4^ Department of Translational Oncology, Genentech, South San Francisco, California 94080, USA; ^5^ Center for Childhood Cancer, The Research Institute at Nationwide Children's Hospital, The Ohio State University College of Medicine, Columbus, Ohio 43205, USA; ^6^ Department of Pediatrics, The Ohio State University College of Medicine, Columbus, Ohio 43205, USA; ^7^ Department of Otolaryngology, The Ohio State University College of Medicine, Columbus, Ohio 43205, USA

**Keywords:** protein kinases, p21-activated kinase, neurofibromatosis, meningioma, signal transduction, small molecule inhibitors

## Abstract

Neurofibromatosis type 2 (NF2) is an autosomal dominant disorder characterized by the development of multiple tumors in the central nervous system, most notably schwannomas and meningiomas. Mutational inactivation of *NF2* is found in 40–60% of sporadic meningiomas, but the molecular mechanisms underlying malignant changes of meningioma cells remain unclear. Because group I p21-activated kinases (Paks) bind to and are inhibited by the *NF2*-encoded protein Merlin, we assessed the signaling and anti-tumor effects of three group-I specific Pak inhibitors - Frax597, 716 and 1036 - in *NF2^−/−^* meningiomas *in vitro* and in an orthotopic mouse model. We found that these Pak inhibitors suppressed the proliferation and motility of both benign (Ben-Men1) and malignant (KT21-MG1) meningiomas cells. In addition, we found a strong reduction in phosphorylation of Mek and S6, and decreased cyclin D1 expression in both cell lines after treatment with Pak inhibitors. Using intracranial xenografts of luciferase-expressing KT21-MG1 cells, we found that treated mice showed significant tumor suppression for all three Pak inhibitors. Similar effects were observed in Ben-Men1 cells. Tumors dissected from treated animals exhibited an increase in apoptosis without notable change in proliferation. Collectively, these results suggest that Pak inhibitors might be useful agents in treating *NF2*-deficient meningiomas.

## INTRODUCTION

Meningiomas arise from arachnoid cap cells located in the arachnoid mater and represent the most common intracranial neoplasia, accounting for approximately 30% of central nervous system (CNS) tumors [1]. According to the World Health Organization classification, approximately 75% and 20% of cases are benign (grade I) and atypical (grade II), respectively. Although only 5% of meningiomas are categorized as anaplastic (grade III), some patients with grade I tumors progress to higher histological grade when they recur [2]. The oncogenic mechanisms involved in the transformation of meningioma cells remain to be elucidated, limiting the opportunity to develop targeted therapies.

Meningiomas have been reported to occur in ~50% of neurofibromatosis type II (NF2) patients and are frequently multifocal, originating either in cranial or spinal locations [3]. Tumors associated with NF2 often display more aggressive histologic features than sporadic meningiomas [4, 5]. However, the frequency of *NF2* mutations is similar in all pathological tumor stages, suggesting that NF2 is important for tumor initiation but not critical for malignant progression. As such, it has been inferred that other factors, such as additional genetic alterations may be responsible for progression within this population.

Aberrations in signaling pathways have been identified in meningiomas and implicated in its tumorigenesis [6, 7]. For example, deregulation of PI3K/Akt signaling has been found to correlate with aggressive behavior of malignant tumors, whereas the Erk pathway is thought to be involved in both proliferation and apoptosis [8]. Molecular studies indicate that p21-activated kinases (Paks), in particular Pak1, are required for the activation of both these pathways in many cell types [9–11]. Paks are serine/threonine protein kinases that act as downstream effectors for the small GTPases Cdc42 and Rac in a variety of cellular processes [12–14]. Pak is known to restrain the tumor suppressor function of Merlin, the protein encoded by the *NF2* gene, via phosphorylation at serine 518 [15, 16]. Reciprocally, Merlin inhibits the interaction between Pak and Rac and plays an inhibitory role in Rac-dependent signaling, and loss of Merlin results in increased Pak activity. These data suggest that there is a mutual negative regulatory loop between Pak and Merlin [17, 18] and that inhibiting Pak might be beneficial in the setting of NF2, as has been demonstrated in NF2-related schwannomas [19–21]. The role of Paks in NF2-related meningioma, however, has not previously been examined.

Here, we show that Pak1 expression is positively correlated with the degree of malignancy in primary meningiomas. Reduction of group I Pak activity by genetic or pharmacological means was associated with a partial G1 cell cycle arrest, decreased motility, and deceleration of meningioma growth in *NF2*-deficient meningioma cell lines. In an orthotopic transplant model, we found that Pak inhibitors were effective in reducing tumor growth of both benign and malignant meningioma *in vivo*. These data suggest that Pak1 represents a reasonable therapeutic target in NF2-related meningioma.

## RESULTS

### Elevated Pak1 expression in meningioma cells

**Figure 1:**
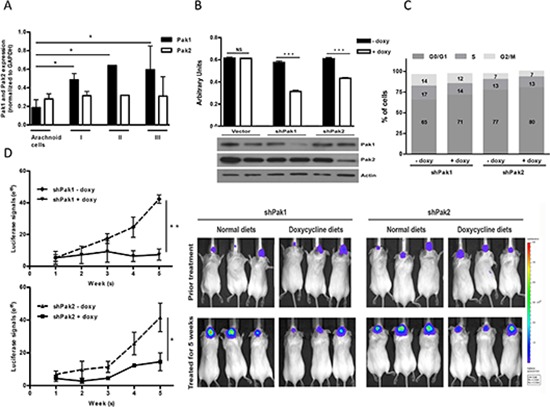
Contribution of Pak1 and Pak2 to cell proliferation and tumor growth in meningioma cells **(A)** Expression of Pak1 and Pak2 were analyzed and quantified based on pathological stages (values are mean ± SEM); Arachnoid cells (*N* = 3), Stage I (*N* = 7), Stage II (*N* = 1) and Stage III (*N* = 2). Immunoblot was shown in Figure S1A. **(B)** Proliferation of KT21 cells after infection with shRNA was measured by MTT assay. Immunoblot analysis showed loss of Pak1 and Pak2 in shRNA-infected cells. **(C)** Cells bearing shPak1 and shPak2 were stained with propidium iodide and subjected to cell cycle analysis by flow cytometry. The data are representative of 3 independent experiments. **(D)** KT21 cells harboring either shPak1 or shPak2 were stereotactically injected at the skull base and the mice were fed with doxycycline diet or normal rodent foods for 5 weeks. Tumor growth was monitored by BLI according to Materials and Methods. *P < 0.05, **P < 0.005, ***P < 0.0005, student's *t*-test. NS, not significant.

The tissue distribution of Paks has been investigated in several studies [22–24] and data using serial analysis of gene expression (SAGE) have revealed that Pak1 and Pak3 are most highly expressed in brain, whereas Pak2 is ubiquitously expressed. However, little data exists regarding the expression of group I Paks in meningiomas. To understand the contribution by which Paks isoforms might be involved in the growth of meningioma cells, we examined a panel of 13 meningioma cells (11 primary and 2 immortalized) and 3 arachnoid cell lines. We found that Pak1 and Pak2 were much more abundant than Pak3 in both meningioma and arachnoidal cells, as determined by immunoblot (Fig. S1A). In addition, we found a marked increased in the expression level of Pak1 in meningioma cells comparing with arachnoid cells (*t*-test, *P* = 0.046; Fig. 1A). In contrast, there was no statistically significant difference in Pak2 expression between meningioma and arachnoidal cells, irrespective of tumor pathological stages (*t*-test, *P* = 0.74). These findings imply that Pak1 expression, but not Pak2 expression, is associated with tumorigenesis in meningiomas.

### Pak1 knockdown reduces meningioma growth

To investigate the significance of Pak1 and Pak2 in meningiomas, we used doxycycline-inducible short hairpin RNA (shRNA) to reduce Pak1 or Pak2 expression, respectively [25]. NF2-null malignant meningioma KT21-MG1-Luc5D cells (hereafter referred to as KT21), were stably transduced with either empty virus or a virus encoding a Pak1 or Pak2 shRNA construct. Upon addition of doxycycline, shRNA-transduced cells displayed markedly reduction in transcriptional and expression level by 75% and 60% for Pak1 and Pak2, respectively (Fig. 1B and Supplementary Fig. S1B). Pak1 shRNA had no effect on Pak2 expression or vice versa. Depletion of either Pak1 or Pak2 resulted in 45% and 29% inhibition of cell viability, respectively, compared with corresponding cells without doxycycline induction (Fig. 1B). Pak1 knockdown cells exhibited a slight increase in G0/G1 phase (65.2% vs. 71.1%; *P* = 0.015), and a corresponding decrease in S phase, whereas Pak2 depletion cells did not affect cell cycle populations (Fig. 1C). Similar results were observed in an *NF2*-deficient benign meningioma cell line, Ben-Men1-LucB, hereafter referred to as Ben-Men, in which expression level diminished by 54% and 58% for Pak1 and Pak2, respectively. Depletion of either Pak1 or Pak2 resulted in 39% and 32% suppression on cell viability as well (Supplementary Fig. S1C, S1D and S1E).

To assess the role of Pak1 and Pak2 *in vivo*, we stereotactically injected shRNA-transduced KT21 cells to the skull base of SCID mice and monitored tumor growth by bioluminescence imaging (BLI). Mice with established KT21 tumors, ~3 weeks post-transplantation, were fed either a normal rodent diet or diet-containing doxycycline for 5 weeks. Immunohistochemical staining of areas of transplant brain showed a positive staining for Pak1 except in shPak1-bearing mice fed with doxycycline diets, which exhibited an almost total absence of Pak1 immunostain (Supplementary Fig. S2A). Similar effects on Pak2 were observed in mice harboring shPak2 cells. These data show that the doxycycline-regulated shRNA constructs function well *in vivo*.

When fed with a normal rodent diet, mice bearing shPak1 or shPak2-transduced cells showed a 6-fold and 8-fold increase, respectively, in BLI signals (Fig. 1D). In contrast, tumors in mice fed with doxycycline diet grew much more slowly (Fig. 1D). Reduction of Pak1 expression had a greater effect than reduction of Pak2 expression, with no net increase in BLI signal over a five-week period, suggesting that, of these two enzymes, Pak1 has a dominant role in the growth of meningioma.

Expression of proliferation marker phospho-histone 3 and the apoptotic marker cleaved caspase-3 was investigated in tumor tissue from the transplanted mice. Knockdown of either Pak1 or Pak2 induced a combination of slightly reduced proliferation and slightly increased apoptosis, with Pak1 knockdown having the greater effect (Supplementary Fig. S2B, S2C).

### Growth inhibition by Pak small-molecule inhibitors

Since knock down of either Pak1 or Pak2 slowed cell growth and tumor growth, we asked whether such inhibitory effects would be observed using Pak small-molecule inhibitors. Two distinct classes of Pak inhibitors – PF3758309, which potently suppresses both group I and group II Paks [26], and three group I-specific Pak inhibitors of increasing specificity (Frax-597 [11, 20], -716 and -1036) (Table 1, Supplementary Table S1, and Supplementary Fig. S3) – were assessed for the effects on cell survival. The three Frax compounds display IC_50_ values towards Pak1 of 10 nM, 2 nM, and 2 nM, respectively, *in vitro*. All three compounds display IC_50_ values towards Pak4 of > 500 nM (data not shown). Ben-Men and KT21 cells were treated with varying concentrations of these inhibitors and the IC50 values were determined following 3 days of treatment (Supplementary Fig. S4). Exposure of meningioma cell lines to these Pak small molecules inhibited cell proliferation in a dose-dependent manner, with benign meningioma cells (Ben-Men) consistently displaying more sensitivity to Pak inhibitors than malignant meningioma cells (KT21) (Table 1 and Supplementary Fig. S4). A Mek inhibitor (PD325901) had no effect on growth of either of these cells, while an Akt inhibitor (GSK690693) impeded the growth of both *NF2^−/−^* meningioma cell lines, but this inhibitory effect was only seen when the compound was used at high doses.

**Table 1:** IC_50_ values of various inhibitors for arachnoid and meningioma cell lines Cells were treated with varying concentrations of inhibitors for 72 hrs.

NF2^+/+^				NF2^−/−^	
Malignancy	Arachnoid cell AC07 (μM)	Benign MN328 (μM)	Malignant MN525 (μM)	Benign Ben-Men-1 (μM)	Malignant KT21-MG1 (μM)
Frax597	1.178	2.807	1.590	0.393	2.927
Frax716	0.288	0.718	0.494	0.218	1.105
Frax1036	1.242	4.239	3.296	1.888	1.991
PF3758309	1.379	3.425	5.319	29.25 (nM)	0.376
PD325901	> 20	> 20	> 20	> 20	> 7.0
GSK690693	> 20	> 20	> 20	3.271	4.235

The IC50 values of *NF2*-null cells indicated that Pak inhibitors could be applied as therapeutic drugs for treatment of NF2 disease in preclinical models. Because only 60% of sporadic meningiomas are associated with *NF2* abnormalities [3, 27], we also asked whether Pak inhibitors would affect Merlin-expressing meningioma cells. An arachnoid cell (AC07) and two *NF2^+/+^* meningioma cell lines MN328 (benign) and MN525 (malignant) were assessed for sensitivity to Pak inhibitors. All cells treated with Pak inhibitors showed a dose-dependent growth inhibition, as observed by light microscope and cell viability assay. Interestingly, benign meningioma cells MN328 were less sensitive to group I selective Pak inhibitors (Frax-597, -716 and -1036), as compared to MN525 and AC07 cells (Table 1). Notably, whereas both *NF2^−/−^* meningioma cell lines (Ben-Men and KT21) were highly sensitive to PF3758309, with IC50 values in the low to mid nM range, *NF2*-expressing meningioma cells showed much less sensitivity to this compound (IC50 3.4 μM and 5.3 μM for MN328 and MN525 cells, respectively).

### Pak inhibitors suppress proliferation and survival of NF2-null meningioma cells

**Figure 2:**
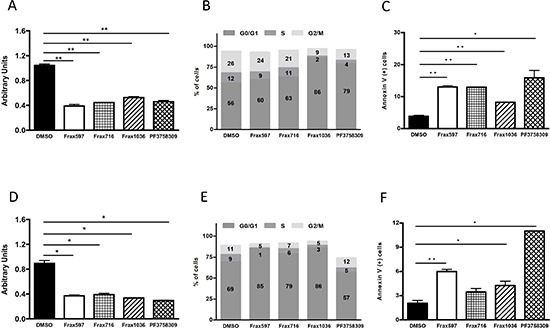
Effects of Pak small-molecules inhibitors on proliferation, cell cycle and apoptosis **(A)** Cell viability analysis of KT21 cells after treatment for 72 hours with Pak inhibitors Frax597 (3 μM), Frax716 (1 μM), Frax1036 (2 μM), or PF3758309 (0.4 μM) by MTT assay. Inhibitor concentrations were chosen based on IC_50_ values (Table 1). **(B)**) Cell cycle analysis of KT21 cells after treatment with Pak inhibitors by FACS. **(C)** Apoptotic analysis of KT21 cells treated with Pak inhibitors. Apoptosis was measured by calculating the percentage of Annexin V positive cells by flow cytometry. **(D)** Cell viability analysis of Ben-Men cells after treatment for 72 hours with Pak inhibitors (Frax597 (0.4 μM), Frax716 (0.2 μM), Frax1036 (2 μM), or PF3758309 (0.03 μM), using MTT assay. **(E)** Cell cycle analysis of Ben-Men cells after treatment with Pak inhibitors by flow cytometry. (**F)** Apoptotic analysis of Ben-Men cells treated Pak inhibitors. Apoptosis was measured by calculating the percentage of Annexin V positive cells by flow cytometry. All data are representative of 3 independent experiments. *P < 0.05, **P < 0.005, student's *t*-test.

Since depletion of Pak1 by shRNA induced a partial G1 cell cycle arrest, we sought to determine whether the same phenomenon would be obtained in *NF2^−/−^* meningioma cells after treatment with small-molecule Pak inhibitors. For these experiments we used two classes of Pak inhibitors: PF3758309, which inhibits all Pak isoforms [11, 26], and Frax-597, -716, and -1036, which inhibit group I Paks only (Supplementary Fig. S3) [11, 20]. In agreement with our shRNA data, each of the four inhibitors suppressed at least 50% on proliferation (Fig. 2A) and induced an increase in the G0/G1 population in KT21 cells (DMSO vs. Frax1036, *t*-test *P* = 0.005; DMSO vs. PF3758309, *t*-test *P* = 0.005; Fig. 2B). A substantial increase in the number of apoptotic cells was also observed after addition of Pak inhibitors to KT21 cells (DMSO vs. Frax597, Frax716, Frax1036, PF3758309, *t*-test *P* = 0.003, 0.001, 0.006 and 0.036, respectively, Fig. 2C). These compounds, with the exception of the pan-Pak inhibitor PF3758309, also suppressed proliferation of Ben-Men cells (Fig. 2D) and induced a significant elevation on G0/G1 population (DMSO vs. Frax597, Frax716, Frax1036, PF3758309, *t*-test *P* = 0.0004, 0.0007, 0.0003 and 0.0003, respectively, Fig. 2E). In the case of PF3758309, there was a general loss of viability accompanied by an increase in sub-G0 cells (Fig. 2E and not shown), consistent with an apoptotic phenotype (DMSO vs. Frax597, Frax716, Frax1036, PF3758309, *t*-test *P* = 0.0009, 0.0657, 0.028 and 0.022, respectively, Fig. 2F).

### Effects of Pak inhibitors on invasiveness

**Figure 3:**
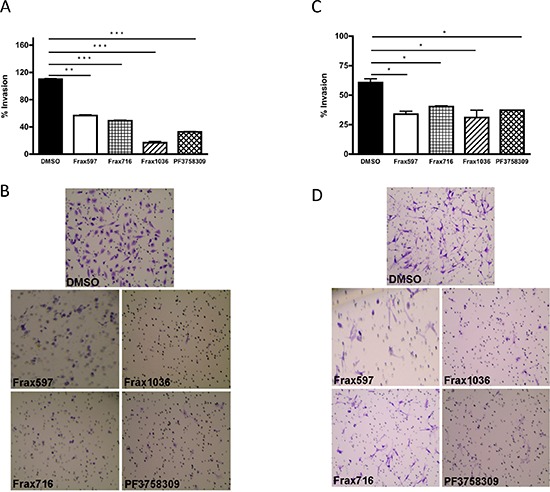
Effects of Pak small-molecules inhibitors on cell invasiveness **(A)** The invasiveness of KT21 cells after treatment with Pak inhibitors was determined by Matrigel invasion chamber assay. Concentrations of inhibitors were as described in Figure 2. The experiments were performed twice with similar results. Data are presented as the mean ± SD of duplicate well measurements from one representative experiment. **(B)** Staining pattern of cells that have migrated and adhered to the bottom surfaces of membranes in cell invasion assay. The small circles are cross-sections of 8 μm pores. **(C)** The invasiveness of Ben-Men cells after treatment with Pak inhibitors was determined by Matrigel invasion chamber assay. The experiments were performed twice with similar results. The data are presented as the mean ± SD of duplicate well measurements from one representative experiment. **(D)** Staining pattern of cells that have migrated and adhered to the bottom surfaces of membranes. The small circles are cross-sections of 8-μm pores. *P < 0.05, **P < 0.005, ***P < 0.0005, student's *t*-test.

We next asked if small molecule Pak inhibitors would affect the invasiveness of *NF2^−/−^* meningioma cells. Ben-Men and KT21 cells were plated in a transwell device, then treated with vehicle or the indicated compounds, and assessed for motility through the membrane. These assays showed that all the Pak inhibitors repressed the invasiveness of benign and malignant *NF2*-null meningioma cells by 70% and ~40%, respectively (Fig. 3).

### Pak inhibition down-regulated multiple signaling pathways

**Figure 4:**
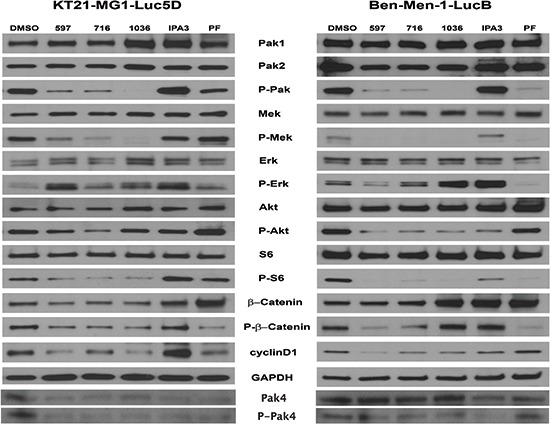
The effect of Pak inhibitors on Erk and Akt-S6 signaling pathways KT21 or Ben-Men cells were treated with inhibitors for 72 hours as described in Figure 2. Following SDS/PAGE and transfer to PVDF membranes, expression levels of Pak, Mek, Erk, Akt, S6, and β-Catenin were assessed by immunoblot using total and phospho-specific antibodies. GADPH was used as loading control.

To better assess the mechanism by which Pak inhibitors affect the proliferation and survival of KT21 and Ben-Men cells, we examined the activation state of several Pak-dependent signaling pathways. Pak1 has been implicated in regulating Erk signaling via phosphorylation of Raf1 and Mek1 [28–30]. As expected, phosphorylation of Pak and Mek1 (at Ser298, the putative Pak phosphorylation site) was dramatically inhibited by Frax597, Frax716 and PF3758309, and nearly abolished by Frax1036 (Fig. 4). Interestingly, despite the loss of group I Pak autophosphorylation and Mek1 Ser298 phosphorylation, none of the compounds except PF3758309 (the least specific inhibitor), caused a decrease in Erk activity. These data suggest that, unlike many other cell types, group I Pak activity is not required for Erk activation in these meningioma cell lines. It is also notable that cells treated with the non-competitive Pak inhibitor IPA3 displayed a paradoxical stimulation of Pak activity, as well as all other signaling molecules measured, rather than inhibition. Such paradoxical effects have been noted by us previously in certain cell types, and may reflect general cellular redox effects of IPA3 [31]. We also made the unexpected observation that in KT21 cells, all of the small molecule Pak inhibitors were associated with decreased expression of the group II Pak, Pak4 (Fig. 4).

Pak1 is thought to affect Akt activation, either via a scaffolding interaction with PDK1 or by direct phosphorylation [9, 32]. Treatment with Pak inhibitors had only a minimal effect on Akt activity in KT21 cells, but a larger effect in Ben-Men cells (Fig. 4). As predicted, down-regulation on ribosomal S6 was noted in both cells following Frax compound addition, indicating decreased mTOR signaling. Interestingly, treatment with PF3758309 did not influence the activity of Akt in either cell type, unlike our previous findings in skin epithelial cells [11].

Cyclin D1 expression, which is important for G1/S progression, was diminished in meningioma cells as a consequence of Pak inhibitor administration (Fig. 4). Strikingly, decreased total and phospho-protein levels of β-catenin were found in KT21 cells after Frax compounds addition. This phenomenon was not found in cells treated with PF3758309, in which only reduction on phosphorylation status was noted. On the other hand, induction on basal level of β-catenin was seen in Ben-Men cells treated with Frax1036, IPA3 and PF3758309. Although Frax597 and Frax716 did not impact expression of β-catenin, phosphorylation of this protein was diminished (Fig. 4). Taken together, these data suggest that inhibition of KT21 cell cycle progression by Pak small molecule inhibitors is mediated, at least in part, by diminished β-catenin signaling with subsequent reduction in cyclin D1 expression.

### Pak inhibitors suppress tumor growth in an orthotopic meningioma model

The pan-Pak inhibitor PF3758309 has been shown to suppress the growth of several Pak1-dependent cancers such as colon, breast, squamous cell, and melanoma [26, 33]. However, this compound inhibits all six Pak isoforms, and thus may have increased toxicities. As Frax597 and its derivatives are orally availability and are specific for group I Paks, we evaluated the effects of these compounds on tumor growth *in vivo*. Luciferase-expressing Ben-Men and KT21 cells, respectively, were implanted into the base of the skull of SCID mice. After tumor establishment, the mice were divided into 4 groups and treated daily with either vehicle or Frax compounds (597, 716 and 1036) for 2–3 weeks and monitored for tumor growth by BLI.

**Figure 5:**
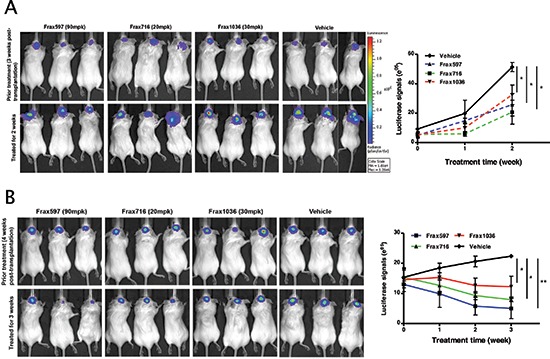
Pak inhibitors suppressed tumor growth in an orthotopic meningioma model Mice bearing tumors were established as described in Materials and Methods and tumor growth was monitored by BLI. **(A)** A representative image showed the bioluminescent signal in the mice bearing KT21 cells, which treated with Pak inhibitors (Frax597, 716 and 1036) and mice treated with vehicle used as a control. Quantitation of bioluminescent signals detected in tumors was conducted from 5 animals per group imaged for 2 weeks. **(B)** A representative image showed the bioluminescent signal in the mice bearing Ben-Men cells, which treated with Pak inhibitors (Frax597, 716 and 1036) and mice treated with vehicle used as a control. Quantitation of bioluminescent signals detected in tumors was conducted from 5 animals per group imaged for 3 weeks. *P < 0.05, **P < 0.005, student's *t*-test.

Untreated mice bearing KT21 transplants displayed ventricular invasion (Fig. S5), whereas Ben-Men grew at the injection site only (not shown), consistent with a previous report [34]. Treatment with Frax597 impaired KT21 tumor growth by 50% compared to vehicle cohort (Fig. 5A). Larger effects were noted in Frax716-treated mice, with reduction on bioluminescence of 60% (*P* = 0.028). However, these animals also showed >10% loss of body weight compared with control littermates (Fig. S6A), an effect not seen in mice treated with Frax597 or Frax1036. Treatment with Frax1036 also resulted in slower tumor growth, with reduction in BMI signals of 37% (*P* = 0.042; Fig. 5A). It should be noted that, when tested in MDCK cells, FRAX1036 has low permeability and is subjected to extensive efflux, and is hence unlikely to have significant blood brain barrier permeability in mice (apparent permeability (P_app_), apical to basolateral = 1 × 10^−6^ cm/s; basolateral to apical = 22.2 × 10^−6^ cm/s; P_app_ ratio = 22.6).

## DISCUSSION

In this study, we show that there is a positive correlation between expression of Pak1 and meningioma tumor grade, and that inhibiting group I Paks, either by knockdown or by small molecule inhibitors, impairs proliferative and survival signaling, and slows the growth of benign and malignant *NF2*-null meningioma cells, both *in vitro* and *in vivo*. These results extend earlier work on the role of Pak signaling in Merlin function in the setting of Schwannomas, showing that these kinases also play a role in meningioma and might therefore represent new therapeutic targets in this disease.

Both Pak1 and Pak2 have previously been individually linked to Merlin function [15, 16, 18, 21, 35]. Our knockdown studies suggest that both group I Pak isoforms play a role in signaling downstream of Merlin (Fig. 1D). These results are in agreement with those of Yi *et al.*, who showed that both Pak1 and Pak2 contributed to the transformed behavior of fibroblasts expressing a dominant negative form of Merlin [21]. Whether these two kinases act on a common set of substrates or have independent functions is not yet known.

Constitutively activation of Erk and PI3K/Akt pathways has been detected in meningiomas [8, 36, 37]. We analyzed the effects of inhibitors of Pak (Frax597, -716, -1036 and PF3758309), Mek (PD0325901) and Akt (GSK690693) on cell viability in benign and in malignant meningiomas. Compared to the Mek or Akt inhibitor, we found that Pak inhibitors were much more effective in reducing cell proliferation *in vitro*, regardless of degree of malignancy and Merlin expression (Table 1). However, loss of Pak activity did not reduce Erk phosphorylation (Fig. 4). These data are consistent with our previous studies, in which a Pak-specific peptide inhibitor was used to block Pak activation in Schwann cells that expressed a dominant negative form of Merlin. In these experiments, Erk remained active despite loss of Pak activity and loss of proliferative potential [19]. Thus, while Paks are required for Erk activation in many cell types, this does not appear to be the case in the setting of *NF2*-deficient schwannomas or meningiomas. Moreover, these data, plus the lack of effect of Mek inhibitors, suggest that Erk activation is not germane to transformation of *NF2*-deficient Schwann cells or meningeal cells.

Previously, we reported that treatment of a K-ras-driven skin cancer mouse model with either the pan-Pak inhibitor PF3758309 or the group I-specific Pak inhibitor Frax597 induced near complete suppression of Pak activity in tumor tissues and had a strong repressive effect on tumor initiation and progression [11]. In the present study, we also evaluated two new group I Pak small-molecule inhibitors, Frax716 and 1036, which represent more Pak-specific derivatives of the parent molecule, Frax597 (Fig. S3). All three group I Pak inhibitors decreased cell proliferation, survival, and motility (Figs. 2 and 3). These compounds were also effective in the setting of orthotopic xenografts (Fig. 5). As Frax1036 has few off-targets (Supplementary Fig. S3), these data, in combination with the shRNA data shown in Fig. 1, suggest that blockade of group I Pak function alone is sufficient to suppress the growth of NF2-deficient benign or malignant meningioma [9, 32]. The relatively lesser effects of such inhibitors in slowing the growth of malignant, as compared to benign, meningioma cells may be related to the maintenance of Akt activity in such cells (Fig. 4).

Treatment of *NF2*-expressing meningioma cells with Mek or Akt inhibitors did not inhibit tumor cell proliferation *in vitro* (IC50 > 20 μM). These data suggest that Merlin status may influence the response to these inhibitors. Apart from mutations or deletions of *NF2*, other genetic alternations are also likely to contribute to responses to targeted agents. Recently, Clark *et al.* reported a genomic analysis of 300 non-NF2 meningiomas and identified oncogenic mutations in *SMO*, *AKT1*, *KLF4* and *TRAF7* [38]. Although mutations in these genes were not analyzed in this study, understanding the mutational spectra in meningioma will undoubtedly be useful in guiding selection of agents for targeted therapies, including those directed against Paks [39, 40].

While surgery remains the primary treatment for meningioma patients, development of effective medical therapies for these tumors, in particular those associated with *NF2* loss, remains an important goal. Recently, Burns and colleagues have reported that AR42, a histone deacetylase inhibitor, arrested meningioma cells growth in G2/M phase and suppressed tumor growth *in vivo* [34]. This agent, like the direct Pak inhibitors reported here, also induced cell cycle arrest and increased apoptosis. Interestingly, another histone deacetylase inhibitor, FK228, has previously been reported to indirectly decrease Pak1 activity and to inhibit the growth of *NF2*-deficient Schwann cells *in vitro* [41]. Whether this phenomenon represents a general function of histone deacetylase inhibitors remains to be determined; if so, there may be a rationale for the combined use of histone deacetylase inhibitors and Pak inhibitors in tumors driven by loss of *NF2*.

## METHODS

### Meningioma cell lysates

Lysates from 11 primary meningioma cells of different WHO grades and 3 arachnoidal cell lines were kindly provided by Dr. Vijaya Ramesh, Harvard Medical School (Boston, MA). Protein concentration was determined, and equal amount of total proteins were separated on SDS-PAGE.

### Cell cultures

The luciferase-expressing *NF2*-deficient benign meningioma cell line, Ben-Men-1-LucB, have been described previously [34], and the luciferase-expressing *NF2*-deficient malignant meningioma cell line, KT21-MG1-Luc5D, were also used (Chang, LS., et al., unpublished data). The Merlin-expressing normal arachnoidal cell line AC07, benign MN328 and malignant MN525 meningioma cell lines were gifts from Dr. Vijaya Ramesh. Cell lines were authenticated by PCR genotyping to confirm *NF2* status. All cell lines were cultured in high-glucose Dulbecco's Modified Eagle Medium (DMEM) supplemented with 10% fetal bovine serum, 2 mM L-glutamine and 100 U/ml penicillin/streptomycin at 37°C in a humidified 5% CO_2_ incubator.

### Retroviral transductions

Inducible shRNA-bearing retrovirus against Pak1 and Pak2 were previously described [42] and oligonucleotides used in this study are as follows: Pak1 shRNA-1 5′-GAT CCC CGA AGA GAG GTT CAG CTA AAT TCA AGA GAT TTA GCT GAA CCT CTC TTC TTT TTT GGA AA-3′; Pak2 shRNA-5 5′-GAT CCC CTG ACA GAG GAG GAG GAT GAT TCA AGA GAT CAT CCT CCT CCT CTG TCA TTT TTT GGA AA-3′.

The ΦNX packaging cell line (Orbigen) was transfected using Lipofectamine 2000 (Invitrogen). Viral supernatants were harvested 48 hr post-transfection and filtered. Meningioma cells were incubated with retroviral supernatant supplemented with 4 μg/ml polybrene for 4 hr at 37°C, and then were cultured in growth media for 48 hr for viral integration. Green fluorescent protein (GFP)-positive infected cells were selected by flow cytometry (GFP).

### RNA isolation and RT-PCR

Subconfluent cells were plated in 6-well plates overnight and treated with doxycycline for 48 hours. Total RNA was isolated using Tri Reagent (Sigma). First-Strand cDNA was synthesized using SuperScript III reverse transcriptase according to manufacture's protocol and standard PCR was performed subsequently.

### Cell viability assay and flow cytometry

Cells were plated at 3 × 10^3^ cells per well in 96-well plates overnight and treated with various concentrations of Pak inhibitors for 72 hours. Cell viability was measured by MTT assay and the half maximal inhibitory concentration (IC_50_) was calculated.

For cell cycle profiles, subconfluent cells were plated in 60 mm plate and treated with inhibitors for 3 days. Cells were collected, washed and fixed in 70% ethanol. After fixation, cells were resuspended in 0.5 ml of a solution containing 1M Tris-HCl pH 8.0, 0.1% Nonidet P-40, 10 mM NaCl, 50 μg/ml propidium iodide and 70 Kunitz units/ml RNase A. DNA content was analyzed using CELLQuest^TM^ software (Becton Dickinson). A minimum of 10,000 events was collected per sample.

For detection of apoptosis, subconfluent cells were plated in 60-mm plate and treated with inhibitors for 3 days. Cells were collected, washed and resuspended in 1x binding buffer with Annexin V-EGFP and propidium iodide (BioVision, CA) and incubated at room temperature for 5 min in dark. Apoptosis was measured using FACS with collecting 10,000 events per sample.

### Protein kinase specificity measurements

Kinase selectivity was assessed using a commercial service that employs a fluorescence-based, coupled-enzyme assay based on the differential sensitivity of phosphorylated and non-phosphorylated peptides to proteolytic cleavage (Invitrogen, Z'-LYTE^®^) [43]. This assay uses recombinant protein kinases and assay mixed optimized for each particular kinase (http://www.lifetechnologies.com/us/en/home/life-science/drug-discovery/target-and-lead-identification-and-validation /kinasebiology/kinase-activity-assays/z-lyte.html).

### Cell invasion assays

Matrigel invasion chamber (BD Biosciences) were rehydrated in serum-free DMEM medium for 2 hr and then placed in 0.75 ml of DMEM medium supplemented with 5% fetal calf serum. Cells at a density of 2 × 10^4^ suspended in 0.5 ml of DMEM, and seeded onto Matrigel chambers. Cells were allowed to migrate for 18 hr. Cells on the upper surface were gently removed with a cotton bud, and cells that had migrated through the 8-μm pores were fixed with 4% paraformaldehyde for 15 min and stained with 0.1% crystal violet for 15 min. Membranes were washed, removed and mounted on a glass slide, and the level of invasion was quantified by visual counting using a microscope with a 20X objective.

### Orthotopic meningioma model

KT21-MG1-Luc5D and Ben-Men1-LucB cells were harvested, washed and resuspended in PBS (0.5 × 10^6^ and 1.0 × 10^6^ cells/mouse in 5 μl). Eight-week-old SCID mice were anesthetized with ketamine and their heads were stabilized in a small animal stereotaxic instrument. A midline sagittal incision was made on the cranial skin. A burr hole was drilled in the skull with coordinated 1.5 mm anterior and 1.5 mm lateral to the right from the bregma and a 5-μl Hamilton syringe loaded with cells was slowly inserted through the burr hole and ~0.5 mm downward to the skull base. The incision was closed using surgical absorbable gut suture.

### Bioluminescence imaging

Mice transplanted with meningioma cells were imaged using Xenogen IVIS Spectrum (Caliper, MA). Mice were injected intraperitoneally with Rediject D-Luciferin Ultra at 150 mg/kg under anesthetized with isofluorane. Luciferase activity was measured and all bioluminescence imaging was conducted when perk signal was reached. Once tumors were established (~3 weeks post-transplantation), mice were divided into groups for shRNA studies or drug treatments.

For shRNA studies, mice were fed either a normal rodent diet or doxycycline foods for 5 weeks. Tumor growth was monitored every week by BLI. Photon emission was quantified using Living Image software (Caliper, MA) and mean luminescence for each group was calculated (*n* = 5 per group).

For drugs treatment, mice were dosed with Pak inhibitors daily for 14–21 days and tumor growth was monitored every week by BLI. Mean luminescence for each treatment groups (*n* = 5) was calculated as described previously.

### Doxycycline administration

Doxycycline-containing food (625 mg/kg; Harlan Laboratories) was administrated to SCID mice in pelleted form with complete grain-based rodent diet, and the control littermates were fed an identical diet, lacking doxycycline.

### Treatment with Pak inhibitors

Frax597 [11, 20] (C29H28ClN7OS; M.W. 558.10) and Frax716 (C30H30ClN7O2; M.W. 556.06 (Afraxis, CA) were formulated in 10% (PEG400: Tween-80: PVP-K30–90:5:5), 15% Vitamin E-TPGS, and 75% of hydroxypropylcellulose (0.5%) in 50 mM citrate buffer (pH 3.0) and administered by oral gavage at 90 mg/kg/day and 20 mg/kg/day, respectively. Frax1036 (C28H32ClN7O; M.W. 518.05) (Afraxis, CA) was formulated in 20% 2-hydroxypropyl-β-cyclodextrin in 50 mM citrate buffer (pH 3.0) and administrated by oral gavage at 30 mg/kg/day. These doses were chosen to provide minimum plasma concentrations of ≥0.5 μM at 24 hours. All treatments were begun when tumors established and were continued for 2 or 3 weeks, at which time the animals were sacrificed and brain tissues taken for analysis.

### *In vitro* permeability assays

Madin-Darby Kidney cells (MDCKI) were obtained from the National Institutes of Health, (Bethesda, MD). Cells were maintained in Dulbecco's Modified Eagle Medium supplemented with 10% FBS, 80 ng/mL colchicine and 5 μg/mL Plasmocin. Cells were harvested with trypsin and seeded on Millipore Millicell-24 well plates at initial concentrations of 2.5 × 10^5^ cells/mL and allowed to grow for 5 days. Cell monolayers were equilibrated in transport buffer (Hank's Balanced Salt Solution with 10 mM Hepes, pH 7.4) for 60 minutes at 37°C with 5% CO2 and 95% relative humidity prior to the experiment. Dose solutions were prepared in transport buffer and consisted of test compounds (10 μM) and the monolayer integrity marker lucifer yellow (100 μM). The dose solutions were added to the donor chambers and transport buffer was added to all receiver chambers. The transport was examined in the apical to basolateral (A-B) and basolateral to apical (B-A) directions. The receiver chambers were sampled (50 μL) at 60, 120, and 180 min and were replenished with fresh transport buffer after the 60 and 120 min samplings. Lucifer yellow permeability was used as a marker of monolayer integrity for the duration of the experiment and was measured using a fluorescence plate reader (ex: 425; em: 530 nm). Compound concentrations in the donor and receiving compartments were determined by LC-MS/MS analysis. The apparent permeability (P_app_) in the apical to basal A-B and basal to apical B-A directions, was calculated as follows: P_app_ = (dQ/dt) • (1/AC_0_), where: dQ/dt = rate of compound appearance in the receiver compartment; A = Surface area of the insert; and C_0_ = Initial substrate concentration at T0. The efflux ratio (ER) was calculated as (P_app_, B-A/P_app_, A-B).

### Brain tissue preparation, histology, immunohistochemistry, and immunoblotting

All transplanted brains were fixed in 4% paraformaldehyde for 48 hr, and dehydrated and embedded in paraffin. Hematoxylin and eosin stained sections were used for diagnostic purposes and unstained sections for immunohistochemical (IHC) studies. IHC was conducted with the following antibodies: rabbit polyclonal antibodies for Pak1 (1:50; 2602), Pak2 (1:100; 2608), cleaved caspase 3 (1:200; 9664), phospho-histone 3 (1:200; 9701), cyclin D1 (1:50; 2978) (Cell Signaling Technology). The evaluation of the IHC was conducted blindly, without knowledge of the treatments. The percentage of caspase 3, phospho-histone 3 and cyclin D1 positive cells was determined by scanning the slides using an Aperio CS Scanscope scanner and nuclear detection software from the same manufacturer.

Immunoblot analyses were carried out on lysates extracted from cells after treatment for 24 hours (inhibitors with concentration of IC50, as detailed in Fig. legends) or 72 hours (doxycycline for shRNA). Protein concentration was determined, and equal amount of total proteins were separated by SDS-PAGE. Antibodies used included those for Pak1 (2602), Pak2 (2608), Pak3 (2609), Pak4 (3242), phospho-Pak4-6 (3241), Mek1 (9122), Erk (9102), phospho-Erk1/2 (pThr202/pTyr204; 9101), Akt (9272), phospho-Akt (pThr308; 9275), S6 (2217), phospho-S6 (pSer235/pSer236; 4857), phospho-β-Catenin (pSer675; 4176), and cyclin D1 (2978) from Cell Signaling Technology; β-Catenin (610154) from BD Transduction Laboratories; and phospho-Pak (Ser141; 44940G) and phospho-Mek (Ser298; 44460G) from Invitrogen. GAPDH and β-actin were used as loading control.

### Statistical analysis

All experiments were performed at least three times. Results were reported as means ± SD. The significance of the data was determined by two-tailed, unpaired Student's *t-*test with *P* < 0.05 considered statistically significant.

## SUPPLEMENTARY FIGURES AND TABLE


