# Supplementary material for: Group I Paks as therapeutic targets in NF2-deficient meningioma
Source: Oncotarget. 2015 Jan 24;6(4):1981–94. doi: 10.18632/oncotarget.2810 (PMC4385830; doi:10.18632/oncotarget.2810)
Supplement: Supplementary file 1 [file oncotarget-06-1981-s001.pdf]

SUPPLEMENTARY FIGURES AND TABLE

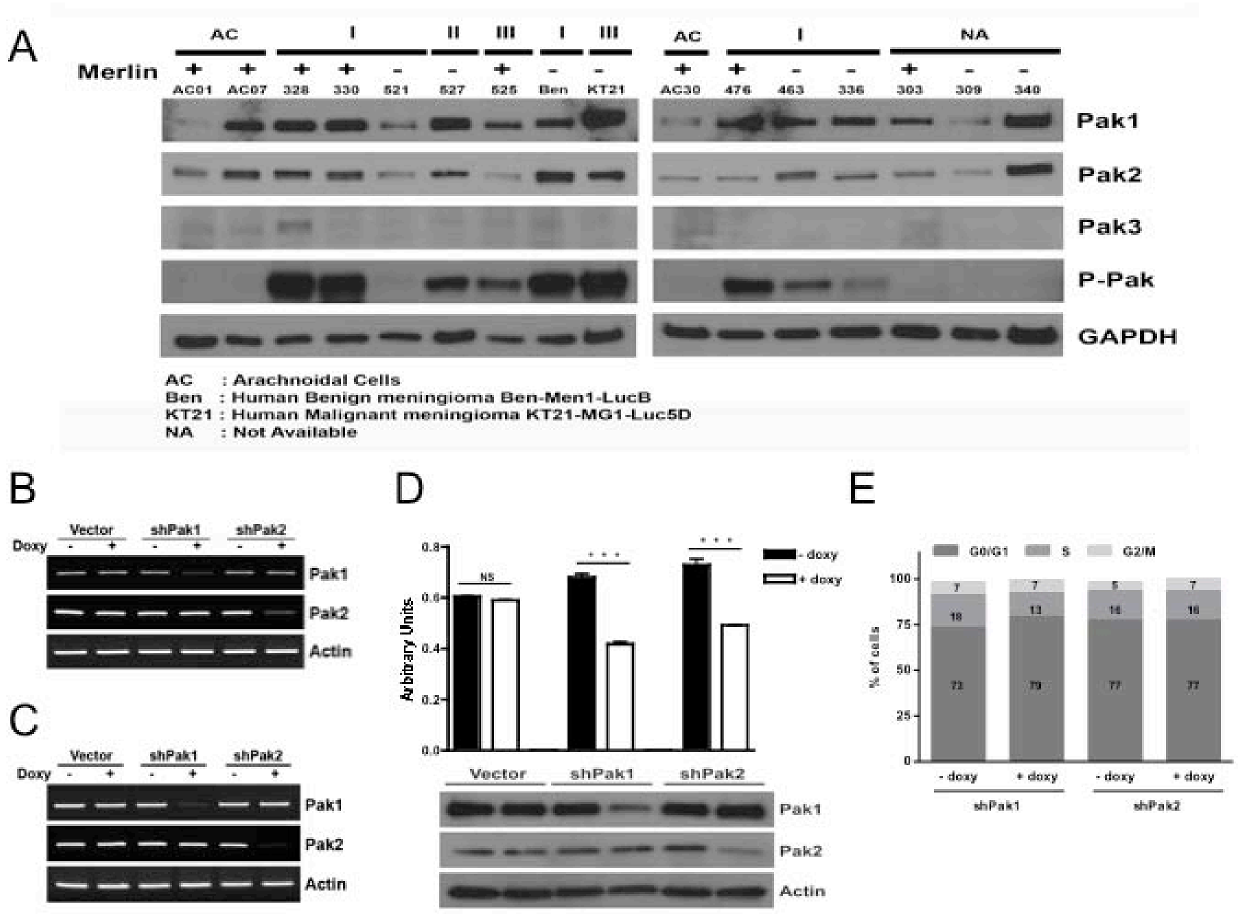

Supplementary Figure S1: Contribution of Pak1 and Pak2 to cell proliferation as well as tumor growth in Ben-Men meningioma cells. **(A)** Immunoblot analysis of a panel of primary meningioma and arachnoidal cells probed with Pak1, Pak2 and Pak3. NA, information not available. **(B)** RT-PCR analysis of *Pak1* and *Pak2* transcripts in shRNA-transduced KT21 cells at 48 hours upon addition of doxycycline. **(C)** RT-PCR analysis of *Pak1* and *Pak2* transcripts in shRNA-transduced Ben-Men cells at 48 hours upon addition of doxycycline. **(D)** Proliferation of Ben-Men cells after infection with shRNA was measured by MTT assay. Immunoblot analysis showed loss of Pak1 and Pak2 in shRNA-transduced cells. **(E)** Cells bearing shPak1 and shPak2 were stained with propidium iodide and subjected to cell cycle analysis by flow cytometry. Data are representative of 3 independent experiments. ***$P < 0.0005$, student's *t*-test. NS, not significant.

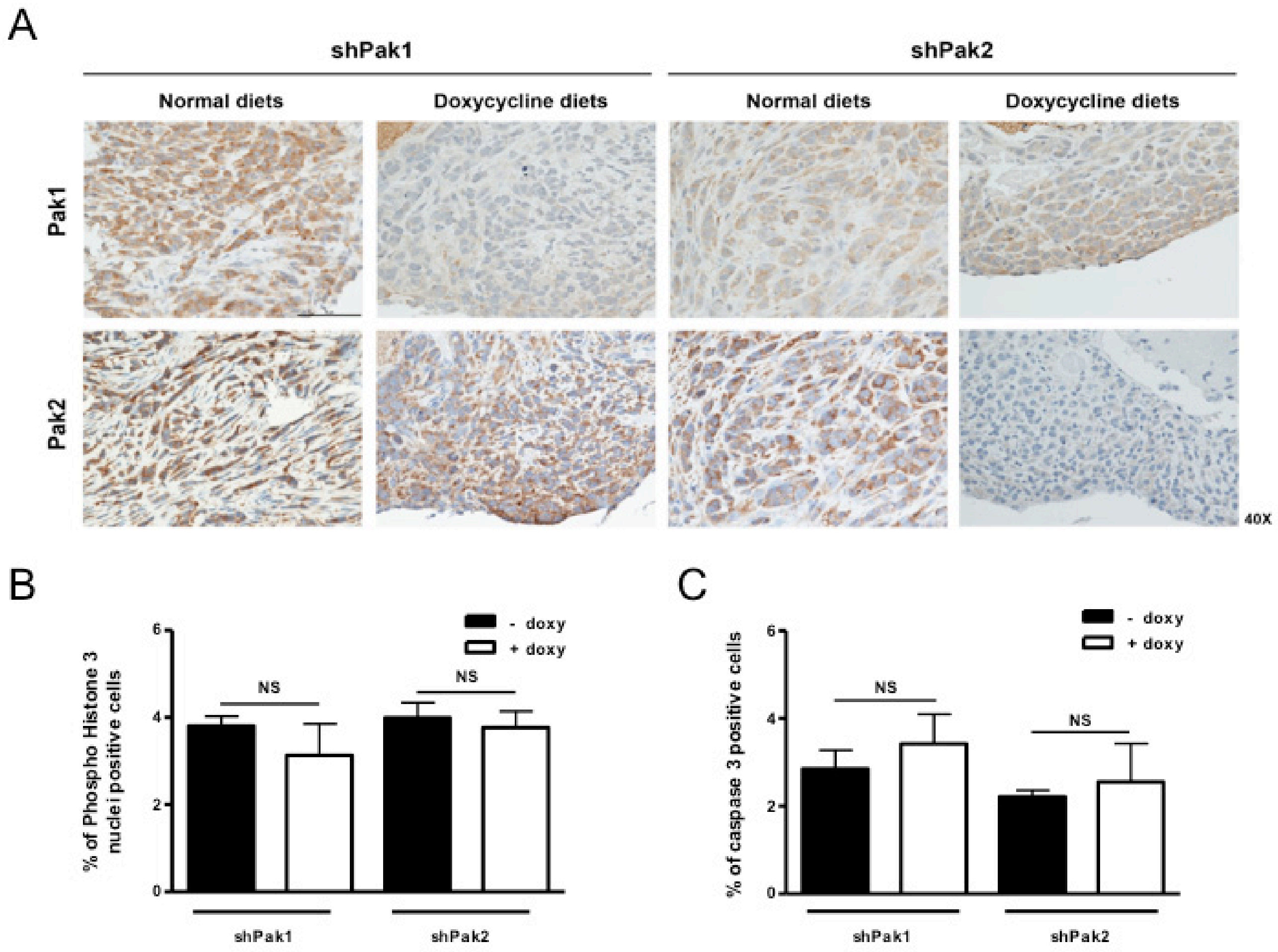

Supplementary Figure S2: Immunohistochemical analysis of brain tissue transplanted with KT21-MG1 meningioma cells carrying either shPak1 or shPak2 after feeding with doxycycline diets or normal rodent foods for 5 weeks. **(A)** Transplanted brain were sectioned and immunostained for Pak1 and Pak2. Scale bar = 100 µm. **(B)** Quantitation of positive cells for phospho-histone 3 was calculated after Aperio Scanscope analysis. **(C)** Quantitation of positive cells for cleaved caspase 3 was calculated after Aperio Scanscope analysis. NS, not significant.

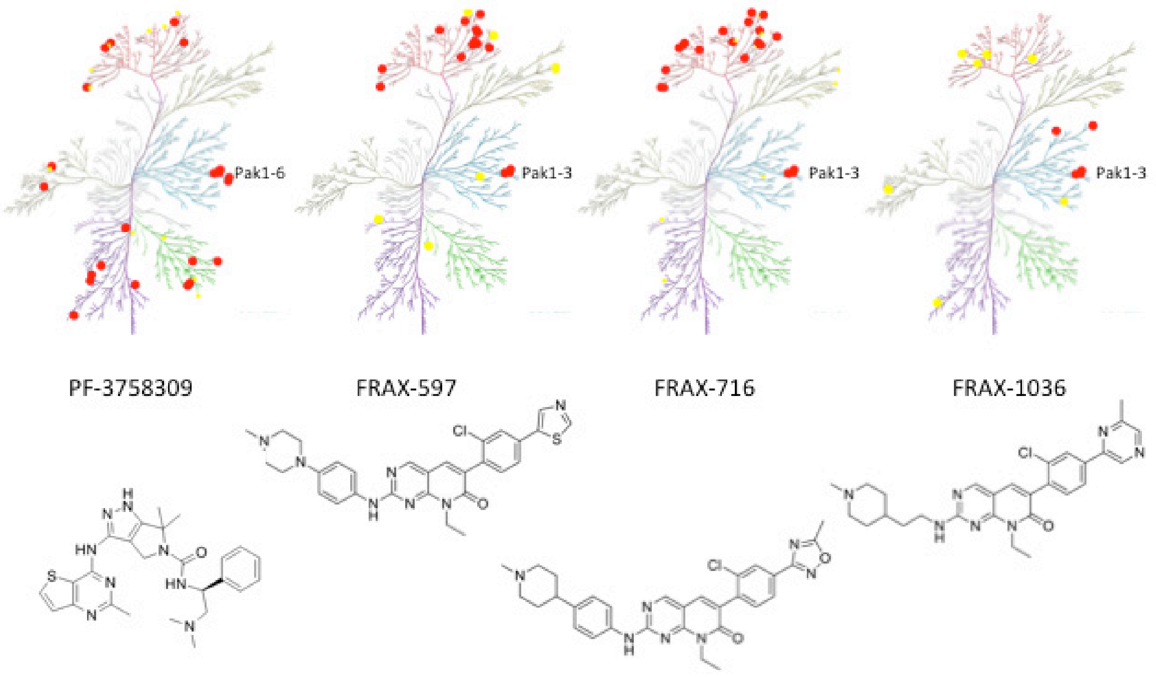

Supplementary Figure S3: Specificity of Pak inhibitors. 1 µg recombinant kinase was incubated at 30°C for 20 min in protein kinase buffer containing 1 µM ATP plus 1 µCi γ-^{32}P-ATP (6000 C_i/mMol), in the presence of 1 µM of the indicated compounds and 1 µg myelin basic protein. The reaction was terminated with 10% TCA and kinase activity was assessed by incorporation of ^{32}P into myelin basic protein. The diagram represents the kinome family tree. Red spheres, >75% inhibition; yellow spheres, >50% inhibition.

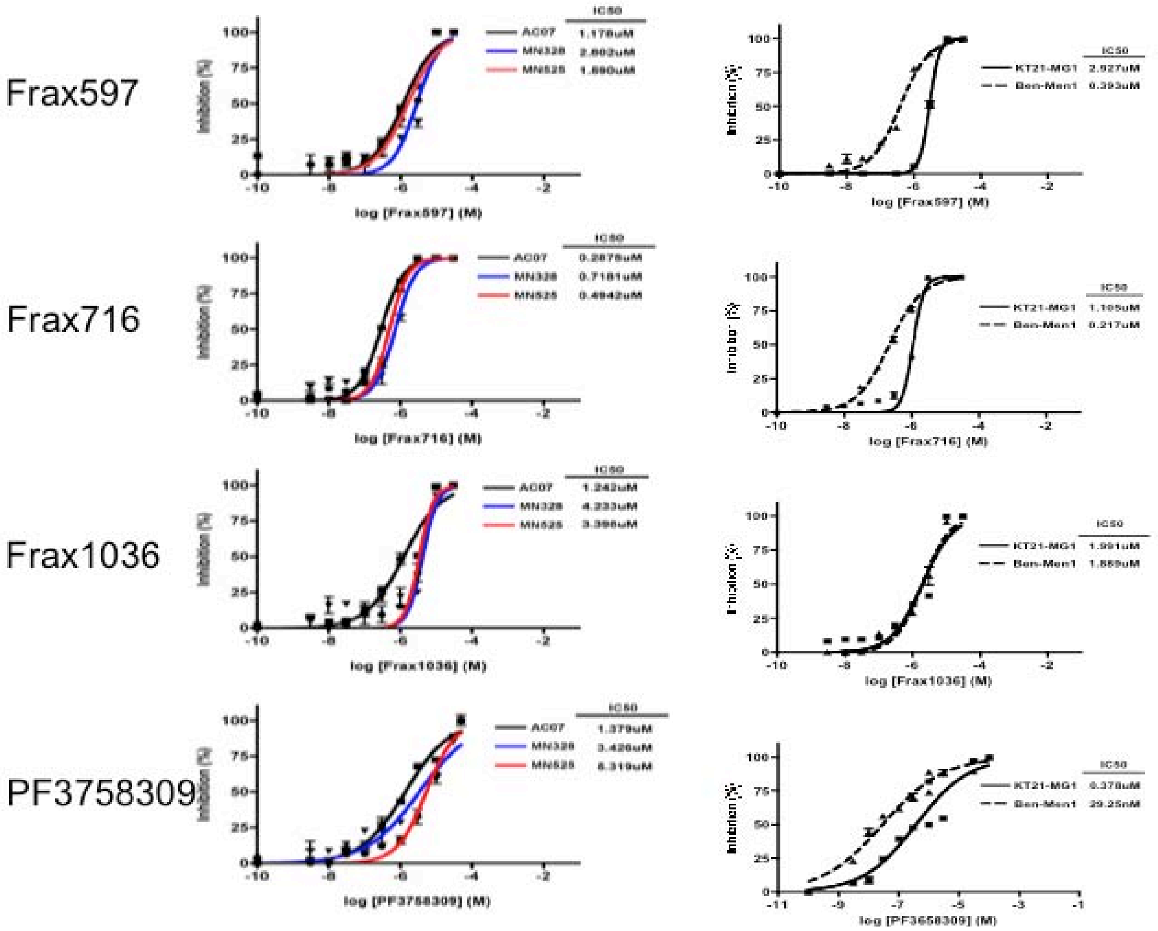

Supplementary Figure S4: Growth inhibition induced by Pak inhibitors. Arachnoid cell (AC07) and meningioma cells were treated with various concentrations of inhibitors for 72 hours, and MTT assay was subsequently performed. Data are presented as a percentage of the control, in which cells were treated with DMSO. Points represent the mean of three independent experiments ± standard error.

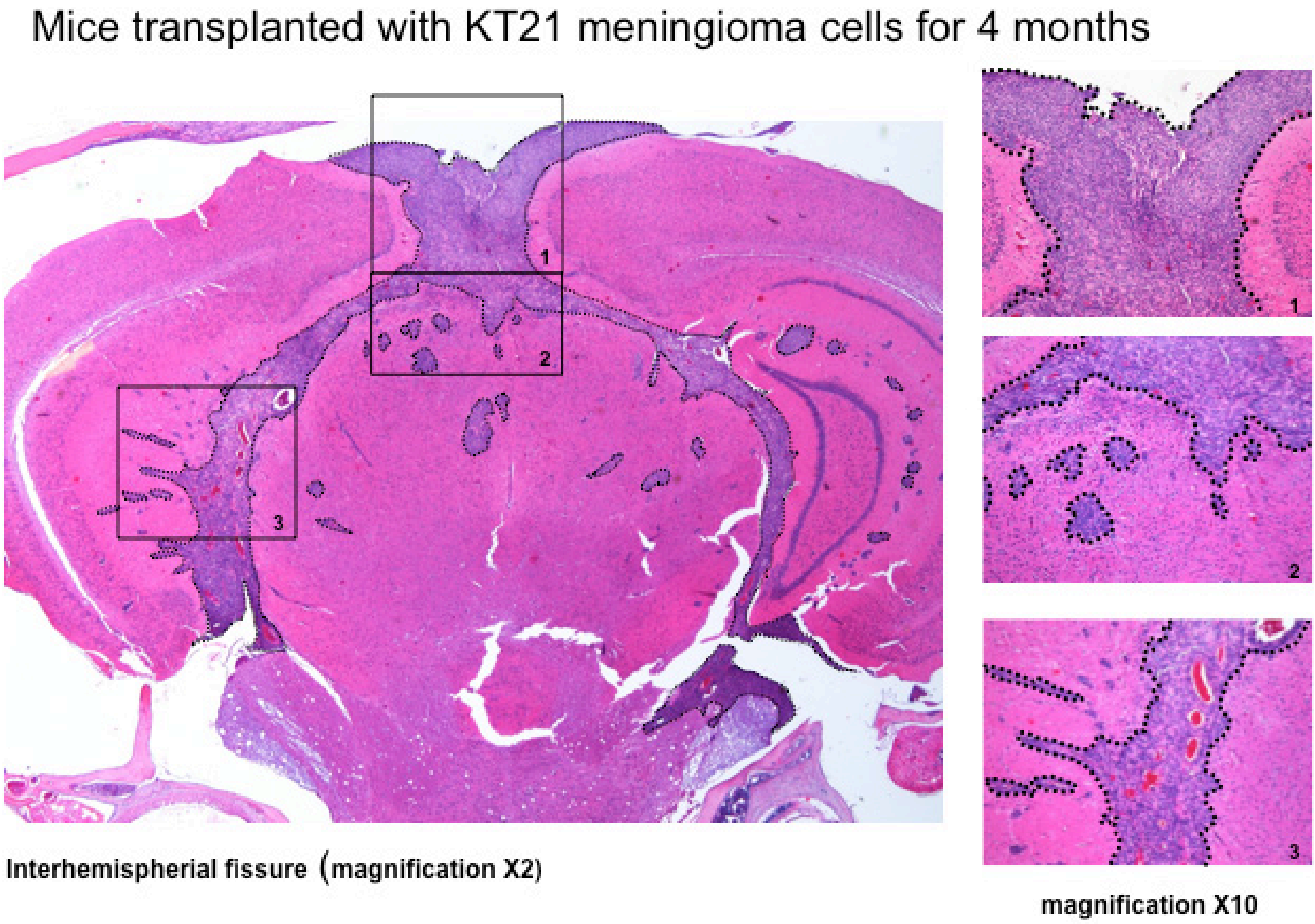

Supplementary Figure S5: Hematoxylin and eosin staining of brain from xenograft-bearing mouse 4 months after transplantation of KT21 meningioma cells.

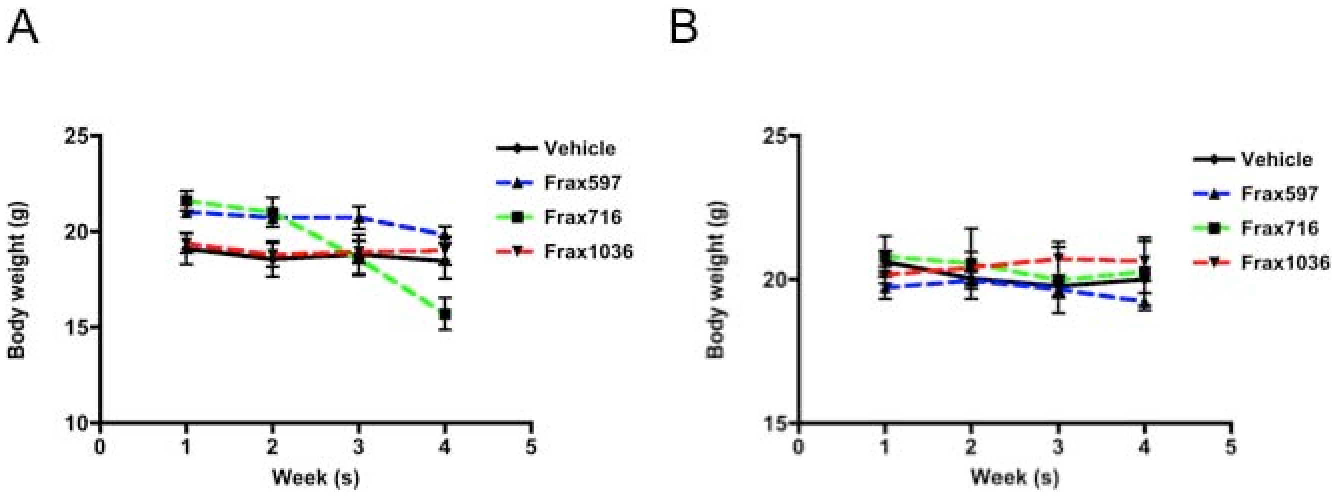

Supplementary Figure S6: Body weight of mice bearing KT21 (A) or Ben-Men (B) cells treated with Pak inhibitors in orthotopic meningioma model.

Supplementary Table S1. % inhibition of activity

		PF3758309	FRAX716	FRAX1036	FRAX1036
Kinase by family	Family				
PAK1	STE	100	95	101	101
PAK3	STE	100	90	106	106
PAK2 (PAK65)	STE	100	100	98	98
PAK4	STE	99	7	6	6
PAK7 (KIAA 1264)	STE	99	17	9	9
PAK6	STE	98	17	27	27
MAP2K1 (MEK1)	STE	39	48	27	27
MAP2K2 (MEK2)	STE	20	79	15	15
CSNK1D (CK1 delta)	CK1	3	6	11	11
CSNK1G2 (CK1 gamma 2)	CK1	9	3	3	3
ADRBK1 (GRK2)	AGC	10	10	0	0
ADRBK2 (GRK3)	AGC	4	2	−1	−1
PDK1 Direct	AGC	67	15	0	0
RP88KA8 (RSK2)	AGC	100	6	11	11
RP88KB1 (p70S6K)	AGC	79	1	10	10
RSK3	AGC			26	26
RSK4	AGC			9	9
AKT2 (PKB beta)	AGC	0	9		
AKT1 (PKB alpha)	AGC	16	1	0	0
PRKCE (PKC epsilon)	AGC	62	14	25	25
PRKCB1 (PKC beta I)	AGC	58	9	18	18
PRKCQ (PKC theta)	AGC	97	12	24	24
PRKACA (PKA)	AGC	100	7	1	1
ROCK1	AGC	5	2	−2	−2
ROCK2	AGC	10	4	7	7
CHEK2 (CHK2)	CAMK	99	17	7	7
MAPKAPK2	CAMK	1	7	0	0
AMPKA1/B1/G1	CAMK	100	13		
AMPKA2/B1/G1	CAMK	100	29		
MARK3	CAMK	100	16	24	24

(*Continued*)

		PF3758309	FRAX716	FRAX1036	FRAX1036
STK22D (TSSK1)	CAMK	90	5	12	12
PIM2	CAMK	4	1	3	3
PIM1	CAMK	6	−4	0	0
CHEK1 (CHK1)	CAMK	91	−2	−14	−14
CDK9/cyclin T1	CMGC	13	3	−8	−8
CDK6/p25	CMGC	18	3		
CDK5/p35	CMGC	15	2	7	7
CDK1/cyclin B	CMGC	12	3	6	6
CDK2/cyclin A	CMGC	22	0	−14	−14
CDK7/cyclin H/ MNAT1	CMGC	96	2	−3	−3
MAPK9 (JNK2)	CMGC	0	20	44	44
MAPK10 (JNK3)	CMGC	5	12	45	45
MAPK8 (JNK1)	CMGC	9	30	79	79
MAPK14 (p38 alpha) Direct	CMGC	7	10	0	0
MAPK11 (p38 beta)	CMGC	8	2	6	6
MAPK1 (ERK2)	CMGC	23	7	5	5
MAPK3 (ERK1)	CMGC	18	8	4	4
G3K3A (G3K3 alpha)	CMGC	52	−9	7	7
G3K3B (G3K3 beta)	CMGC	77	14	0	0
DYRK1A	CMGC	6	3	5	5
DYRK1B	CMGC	7	13		
FRAP1 (mTOR)	ATYPICAL	0	1	0	0
P1K3CA/PIK3R1 (p110 alpha)	ATYPICAL	0	−1	0	0
P1K3CA (p110 gamma)	ATYPICAL	0	0	0	0
P1K3C2B (P13K-C2-beta)	ATYPICAL	8	−1	2	2
SPHK1	ATYPICAL	4	−3	0	0
ABL1	TK	62	93	18	18
ABL2 (Arg)	TK	77	82		
BTK	TK	74	87	5	5
ITK	TK	23	84	0	0
LYNA	TK	76	88	39	39

(*Continued*)

		PF3758309	FRAX716	FRAX1036	FRAX1036
SRC	TK	74	97	16	16
YES1	TK	86	89	18	18
EPHA2	TK	18	68	18	18
PTK1 (FAK)	TK	38	22	9	9
SYK	TK	32	21	4	4
ZAP70	TK	10	8	3	3
ALK	TK				
INSR	TK	7	14	0	0
IGF1R	TK	10	23	7	7
NTRK1 (TRKA)	TK	73	92		
MST1R (RON)	TK	8	40	55	55
MET (cMet)	TK	19	38	36	36
AXL	TK	67	45	21	21
RET	TK	81	91	8	8
FGFR2	TK	72	60	15	15
FGFR3	TK	31	35	0	0
FGFR4	TK	11	20	9	9
FLT1 (VEGFR1)	TK	5	37	14	14
KDR (VEGFR2)	TK	20	90	16	16
CSF1R (FMS)	TK	20	97	41	41
KIT	TK	7	77	32	32
FLT3	TK	78	92	10	10
PDGFRA (PDGFR alpha)	TK	2	95	2	2
PDGFRB (PDGFR beta)	TK	14	82	83	83
TEK (Tie2)	TK	0	98	2	2
EGFR (ErbB1)	TK	4	20	2	2
ERBB2 (HER2)	TK	4	8	2	2
ERBB4 (HER4)	TK	2	77	0	0
JAK1	TK	2	1	1	1
TYK2	TK	14	54	6	6
JAK2	TK	14	7	0	0
JAK3	TK	10	3	0	0
RAF1 (cRAF) Y340D Y341D	TKL	26	74	11	11
BRAF	TKL	52	72	7	7
ACVR1B (ALK4)	TKL	1	−9	0	0
